# Mendelian Disorders in an Interstitial Cystitis/Bladder Pain Syndrome Cohort

**DOI:** 10.1002/ggn2.202200013

**Published:** 2022-11-27

**Authors:** Elicia Estrella, Shira Rockowitz, Marielle Thorne, Pressley Smith, Jeanette Petit, Veronica Zehnder, Richard N. Yu, Stuart Bauer, Charles Berde, Pankaj B. Agrawal, Alan H. Beggs, Ali G. Gharavi, Louis Kunkel, Catherine A. Brownstein

**Affiliations:** ^1^ Department of Neurology Boston Children's Hospital Harvard Medical School Boston MA 02115 USA; ^2^ Division of Genetics and Genomics Boston Children's Hospital Harvard Medical School Boston MA 02115 USA; ^3^ The Manton Center for Orphan disease Research Boston Children's Hospital Harvard Medical School Boston MA 02115 USA; ^4^ Research Computing Information Technology Boston Children's Hospital Boston MA 02115 USA; ^5^ Department of Urology Boston Children's Hospital Boston MA 02115 USA; ^6^ Department of Anesthesiology, Critical Care and Pain Medicine Boston Children's Hospital Boston MA 02115 USA; ^7^ Department of Anaesthesia Harvard Medical School Boston MA 02115 USA; ^8^ Division of Newborn Medicine Boston Children's Hospital Harvard Medical School Boston MA 02115 USA; ^9^ Institute for Genomic Medicine Vagelos College of Physicians & Surgeons Columbia University New York NY 10032 USA; ^10^ Division of Nephrology Department of Medicine Vagelos College of Physicians & Surgeons Columbia University New York NY 10032 USA; ^11^ Center for Precision Medicine and Genomics Department of Medicine Vagelos College of Physicians & Surgeons Columbia University New York NY 10032 USA

**Keywords:** bladder, genetics, genital, genomics, Mendelian, pain, urinary

## Abstract

Interstitial cystitis/bladder pain syndrome (IC/BPS) is a chronic pain disorder causing symptoms of urinary frequency, urgency, and bladder discomfort or pain. Although this condition affects a large population, little is known about its etiology. Genetic analyses of whole exome sequencing are performed on 109 individuals with IC/BPS. One family has a previously reported *SIX5* variant (ENST00000317578.6:c.472G>A, p.Ala158Thr), consistent with Branchiootorenal syndrome 2 (BOR2). A likely pathogenic heterozygous variant in *ATP2A2* (ENST00000539276.2:c.235G>A, p.Glu79Lys) is identified in two unrelated probands, indicating possible Darier‐White disease. Two private heterozygous variants are identified in *ATP2C1* (ENST00000393221.4:c.2358A>T, p.Glu786Asp (VUS/Likely Pathogenic) and ENST00000393221.4:c.989C>G, p.Thr330Ser (likely pathogenic)), indicative of Hailey‐Hailey Disease. Sequence kernel association test analysis finds an increased burden of rare *ATP2C1* variants in the IC/BPS cases versus a control cohort (*p* = 0.03, OR = 6.76), though does not survive Bonferroni correction. The data suggest that some individuals with IC/BPS may have unrecognized Mendelian syndromes. Comprehensive phenotyping and genotyping aid in understanding the range of diagnoses in the population‐based IC/BPS cohort. Conversely, *ATP2C1, ATP2A2*, and *SIX5* may be candidate genes for IC/BPS. Further evaluation with larger numbers is needed. Genetically screening individuals with IC/BPS may help diagnose and treat this painful disorder due to its heterogeneous nature.

## Graphical abstract

Exome data for 109 individuals with interstitial cystitis/bladder pain syndrome is analyzed for Mendelian disorders. Two genes identified within the cohort, *ATP2C1* and *ATP2A2*, are associated with dermatological phenotypes. Further evaluation with larger numbers is needed, though genetically screening individuals with IC/BPS may be useful in diagnosing and treating this painful disorder due to its heterogeneous nature.

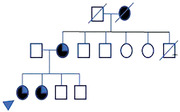

## 1 Introduction

Interstitial cystitis/bladder pain syndrome (IC/BPS) is a chronic pain disorder causing symptoms of urinary frequency, urgency, and bladder discomfort or pain.^[^ ^1^ ^]^ Prevalence estimates in the past decade now exceed 10 million in the United States (3–7% of women and 2–4% of men).^[^ ^2^, ^3^ ^]^ Symptoms of IC/BPS are often debilitating and can affect work, family, interpersonal relationships, sleep, and sexual activity.^[^ ^4^, ^5^ ^]^

Understanding the etiology and pathogenesis of interstitial cystitis is paramount to the development of targeted therapies against this debilitating condition. The average time from presentation to the general practitioner to specialty clinic referral and subsequent diagnosis ranges from 3 to 7 years.^[^ ^6^ ^]^ The diagnosis of IC/BPS is associated with high costs; while IC/BPS accounted for only 0.1% of all medical treatment and prescription drug claims in 2002 in the United States. That year the mean annual medical cost for individuals diagnosed with IC/BPS was $8420, compared to a mean of $4169 for those without IC/BPS.^[^ ^7^ ^]^

Recent studies of kidney and urologic disorders have shown that many individuals have genetic diseases that remain undiagnosed after standard clinical evaluation.^[^ ^8^, ^9^, ^10^, ^11^, ^12^ ^]^ For example, 5–8% of individuals with developmental disorders of the urinary tract have an undetected genomic disorder.^[^ ^13^, ^14^, ^15^ ^]^ We also have seen reports of increased incidence of IC/BPS in first degree relatives of people diagnosed with IC/BPS.^[^ ^16^ ^]^ Together this data point to a genetic etiology for IC/BPS.

Our hypothesis was that some individuals with IC/BPS had undiagnosed Mendelian conditions, and that the genes behind these Mendelian conditions could be informative candidate genes leading us to an IC/BPS gene or gene pathway and ultimately an etiology. Therefore, we screened biological samples and phenotypic information on 109 unrelated individuals with severe IC/BPS. To test for Mendelian causes of IC/BPS, a diagnostic variant analysis of exome sequencing (ES) data was performed. To test whether the candidate genes identified in the Mendelian analysis had increased rare variant burden, we used the Genomic Learning System at Boston Children's Hospital (Boston, MA) to perform sequence kernel association test (SKAT) analysis implemented in the Genuity Science Sequence Miner platform (Cambridge, MA).

## 2 Experimental Section

### 2.1 Phenotyping and Diagnosis of Cases

238 individuals with IC/BPS were identified by collaborating physician referral or self‐referral from ads placed on Clinicaltrials.gov, the Interstitial Cystitis Association (ICA) website and newsletters, and Interstitial Cystitis Network (ICN). Inclusion criteria were 1) Diagnosed with IC/BPS by a clinician 2) Symptoms of urinary urgency and pelvic, suprapubic and/or abdominal pain for 3 months or longer in a 6 month period. Exclusion criteria were 1) evidence of bacterial urinary tract infections in last 3 months, 2) structural urinary tract abnormalities, and 3) bladder, prostate, cervical or uterine cancer. Probands included both adults and children with an IC/BPS diagnosis. All individuals were enrolled in the research program following informed consent under an IRB‐approved protocol at Boston Children's Hospital (04‐11‐160).

Each proband with a clinical diagnosis of IC/BPS in the cohort has had the following clinical workup: cystoscopy with hydrodistension (i.e., looking for glomerulations, Hunner's ulcers, and cancer), a physical exam that ruled out structural defects and infection via urinalysis, and a voiding analysis to assess for muscle dysfunction (e.g., detrusor or pelvic floor). Participants have also completed several questionnaires including the O'Leary‐ Sant for women,^[^ ^17^ ^]^ the National Institutes of Health Chronic Prostatitis Symptom Index (NIH‐CPSI) for men,^[^ ^18^ ^]^ and a diagnostic questionnaire that asks about symptoms and co‐morbidities common in IC/BPS. Before enrollment, all data were reviewed by the study urologist to confirm the IC/BPS diagnosis.

After enrollment, a 3–4 generation pedigree focusing on urinary and pain symptoms was obtained. Additional family members were enrolled using a tiered priority system. Families were stratified based on factors like presence of Hunner's ulcers (tier 1, *N* = 10 families), multiple family members with IC/BPS diagnosis (tier 2, *N* = 45 families), multiple family members with IC/BPS symptoms (tier 3, *N* = 60 families), and sporadic cases and family members (tier 4, *N* = 123 individuals and families). A review of the inheritance pattern in this cohort revealed an autosomal dominant inheritance pattern with variable expressivity in more than 50% of enrolled families. In total, 415 individuals were enrolled. 177 additional family members from 135 families were recruited, including 121 family members with a diagnosis of IC/BPS and 56 without a diagnosis of IC/BPS. 103 individuals did not have any relative enroll.

### 2.2 Exome and Mendelian Analyses

Saliva samples were collected and white blood and buccal epithelial cell DNA were extracted using Oragene kits (DNA Genotek). The highest quality DNA for the cohort was sent for ES; 109 affected individuals (probands or an affected family member) from 109 distinct families were selected. Tiers, age of onset, and questionnaire scores were not taken into account as all individuals had a diagnosis of IC/BPS. Illumina‐based short read whole ES was conducted at the Broad Institute (Cambridge, MA) as previously described.^[^ ^19^, ^20^ ^]^

After ES, all variants were annotated based on their predicted effect on protein function using the Codified Genomics platform (San Diego, CA). A filter on MAF of 0.005 for autosomal dominant and 0.01 for autosomal recessive variants, was chosen as a reasonable maximum allele frequency for disease‐causing variants. Allelic frequencies were estimated based on public databases including gnomAD, dbSNP, and the 1000 Genomes Project, as well as 2672 internal control sequences (healthy parents of individuals with suspected Mendelian disorders). Prediction of pathogenicity was computed using algorithms including PolyPhen.^[^ ^20^, ^21^ ^]^

Additional resources for facilitating variant interpretation and establishing genetic causality included the Human Gene Mutation Database (HGMD), Online Mendelian Inheritance in Man (OMIM), and an in‐house curated list of genes and pathways with a known or postulated association to IC/BPS, pain, bladder, kidney, urogenital, immunological, skin and developmental disorders. Both pleiotropic and non‐pleiotropic genes were included as candidates.

Next, the potentially disease‐causing variants in each individual were assessed using automated ACMG criteria scoring in Varsome (varsome.com) and manually reviewed for accuracy of clinical interpretation.^[^ ^22^ ^]^ Finally, Variants that were classified as “Variants of Uncertain Significance (VUS),” “Likely Pathogenic,” and “Pathogenic” were manually queried for ACMG classification and for presence in ClinVar.

All individuals were also called with the Genome Analysis Toolkit following the best practice workflow as described in Rockowitz et al.^[^ ^20^ ^]^ and resulting VCF data analyzed in the Genuity Science (Reykjavik, Iceland). Within Genuity, ES data were analyzed for potentially disease causing variants in each proband. Again, a filter on MAF of 0.005 for autosomal dominant and 0.01 for autosomal recessive variants was used. Variant Effect Prediction (VEP) high/moderate (i.e., transcript ablation, splice acceptor variant, splice donor variant, stop gained, frameshift variant, stop lost, start lost, transcript amplification, inframe insertion, inframe deletion, missense variant, protein altering variant, splice region variant, or incomplete terminal codon variant) were considered. Genuity automatically screens for presence in ClinVar, HGMD, and OMIM.

### 2.3 Statistical Analysis

SKAT is a supervised, flexible, computationally efficient regression method to test for association between genetic variants (common and rare) in a region and a continuous or dichotomous trait while adjusting for covariates. The authors were interested to learn about the gene burden for the genes identified as variants of uncertain significance, pathogenic, or likely pathogenic by the Mendelian analysis/Varsome pipeline described above.

#### 2.3.1 Preprocessing of Data

109 individuals (93 female and 16 male) with IC/BPS were loaded as cases for the SKAT analysis. Cases with diagnostic variants were removed (6 individuals removed), along with ES with poor coverage, abnormal average SNV reference fraction, or yield below 0.80 (17 individuals removed). The yield field indicates the proportion of the overall variants that can be assigned to either homozygous reference or heterozygous call. If a mix of ES and WGS or different capture kits was used, the ES samples will show a very low yield as coverage was missing in areas.

After filtering, 86 affected individuals remained (80 female, 6 male). Controls consisted of 90 unrelated healthy individuals (80 females, 10 males), who were unaffected parents of patients with suspected Mendelian disorders unrelated to IC/BPS. Controls were 100% Caucasian (self‐reported), matching the 100% Caucasian background of this subset of the IC/BPS cohort.

#### 2.3.2 Gene Selection

Five genes were selected based on the results of the Mendelian analysis. Two genes were ATPase calcium‐transporters with pathogenic rare or private variants identified in the cohort: *ATP2C1* and *ATP2A2*. Three additional genes, *SIX5*, *ENAM*, and *DCAF8*, were included due to previously reported pathogenic variants identified in the cohort and expression occurring in the urogenital tissues.

#### 2.3.3 Statistical Methods

SKAT analysis^[^ ^23^ ^]^ was performed in Genuity Science (version 2.0.1).^[^ ^24^ ^]^ VEP high/moderate variants were used for analysis. Allele frequency upper threshold was 0.01, with a Harvey‐Weinberg equilibrium threshold of 1e‐6. Bonferroni correction was applied for five genes tested and a *p*‐value less than 0.01 was considered to indicate significant difference.

Though cases and controls were matched on sequencing platform, sequencing facility, and ancestry, five sets of housekeeping genes were also used as controls to ensure there were no batch effects creating systematic differences between case and control sequences (e.g., increased rare variant calls in IC/BPS cohort). The five sets of five housekeeping genes were selected by a pseudorandom number generator from a list of 3803 housekeeping genes from Eisenberg et al.^[^ ^25^ ^]^ (see Table S1, Supporting Information for housekeeping gene list).

## 3 Results

### 3.1 Demographics

109 unrelated individuals (93 women 16 men) underwent ES. 100% of individuals were Caucasian/White and non‐Hispanic ancestry (self‐reported). Average age and selected descriptive characteristics are stated in **Table** **1**. No children under 18 were sequenced.

**Table 1:** Selected characteristics for the IC/BPS individuals who underwent ES

Gender		N	Min	Max	Mean	Std Dev
**Male**	Years between first symptom and diagnosis	16	0	29	7	9
	Age (years) IC symptoms began	16	0	74	31	17
	Age IC was diagnosed	16	8	74	38	17
	Current age	54	24	77	51	14
**Female**	Years between first symptom and diagnosis	93	0	59	8	10
	Age (years) IC symptoms began	93	2	74	32	16
	Age IC was diagnosed	93	5	75	40	14
	Current age	54	24	81	51	18

### 3.2 Mendelian Analysis

We identified multiple candidate genes with variants of interest across the IC/BPS cohort. Two private heterozygous variants were identified in the ATPase calcium‐transporting type 2C member 1 gene (*ATP2C1)* (ENST00000393221.4:c.2358A>T, p.Glu786Asp, (VUS/Likely Pathogenic) and ENST00000393221.4:c.989C>G, p.Thr330Ser (Likely Pathogenic). (**Table** **2**). These variants were identified in two IC/BPS cohort probands (SF368‐II‐2‐P2 & SF380‐II‐1‐P2). The variant in SF380 was confirmed in CLIA certified genetic testing laboratory. The variant in SF368 is in process of CLIA confirmation. Pathogenic variants in *ATP2C1* are causative for autosomal dominant Hailey‐Hailey disease (OMIM # 169600), a blistering skin disorder.

**Table 2:** Rare and predicted damaging variants in *ATP2C1*, *ATP2A2*, and *SIX5* in IC/BPS

Gene	Associated condition	Location	Change	Number affected, number of families	ACMG categorization of variant	ClinVar classification (Number entries for each classification, accessed Dec 30, 2021)
*ATP2C1*	Hailey‐Hailey	chr3:130716462	ENST00000393221.4: c.2358A>T, p.Glu786Asp,	1,1	VUS/likely pathogenic PM2, PP2, PP3 supporting	N/A
*ATP2C1*	Hailey‐Hailey	chr3:130678173	ENST00000393221.4: c.989C>G, p.Thr330Ser	2,1	Likely Pathogenic PM2 Strong, PP2, PP3 Supporting	N/A
*ATP2A2*	Darier‐White	chr12:110729840	ENST00000539276.2: c.235G>A, p.Glu79Lys	4,2	Likely Pathogenic PM2 Strong, PP2, PP3 Supporting	N/A
*SIX5*	Branchiootorenal syndrome 2 (BOR2)	chr19:46271631	ENST00000317578.6: c.472G>A, p.Ala158Thr	1,1	VUS/likely pathogenic PP5 Strong, PP3 Supporting	VUS (1), Pathogenic (2)

**Figure 1:**
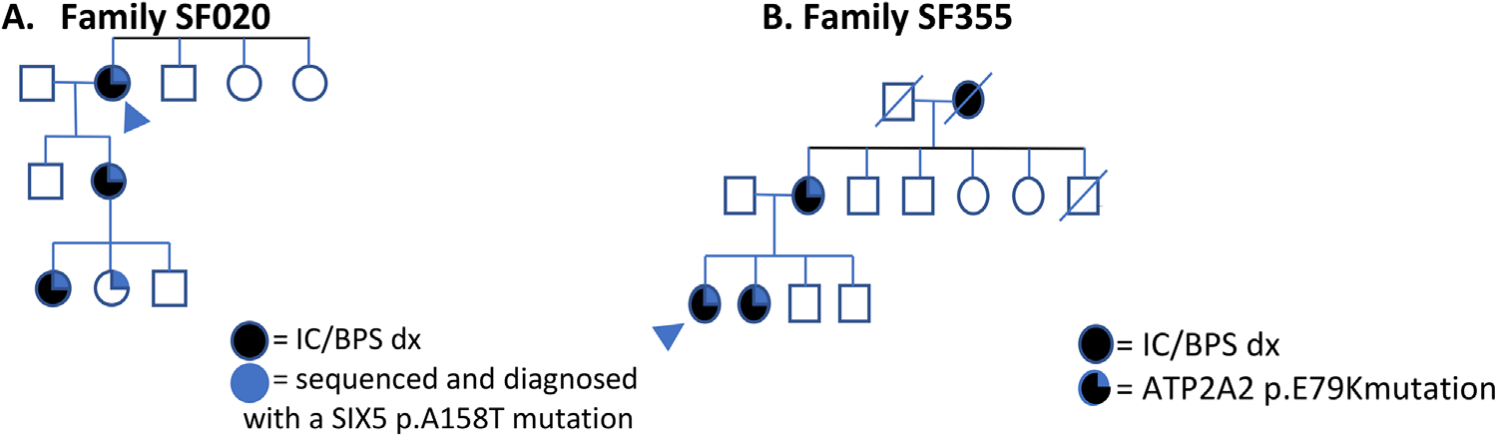
Pedigrees for *SIX5* and *ATP2A2* families.

A VUS/Likely Pathogenic variant in *ATP2A2* (ENST00000539276.2:c.235G>A, p.Glu79Lys) was identified in two probands from the IC/BPS cohort, SF138‐III‐1‐P2, and SF355‐III‐1‐P2. This variant is not identified in gnomAD (accessed September 1, 2022), indicating that the variant is rare or private. This variant is also conserved across 46 vertebrate species.^[^ ^26^ ^]^ CLIA confirmation is ongoing in these participants and affected family members (See **Figure** **1** for Pedigree). The *ATP2A2* gene has been shown to cause Darier‐White disease (OMIM # 124200), another autosomal dominant dermatological disorder. Interestingly, all affected individuals reported skin findings of psoriasis and/or eczema as a comorbidity to their IC/BPS disease.

We also identified a family with a heterozygous variant in the *SIX5* (ENST00000317578.6:c.472G>A, p.Ala158Thr) (See Figure 1 for Pedigree). This variant is previously reported as likely pathogenic, indicating a possible diagnosis of Branchiootorenal syndrome 2 (BOR2, OMIM # 610896). While the variant carriers do not have documented uro‐genital malformations, as they were exclusionary criteria for our study, it is possible that the individuals have subtle urogenital anomalies that eluded detection. Interestingly, one family member was excluded due to a urogenital malformation. She was subsequently enrolled in this study and was confirmed to also harbor the heterozygous *SIX5* variant. We feel this family has a likely diagnosis of BOR2 syndrome with variable expressivity. This variant is in the process of being CLIA confirmed and reported back to family members for further investigation.

A previously reported heterozygous variant in the *DCAF8* gene was identified in an individual (ENST00000368073.3:c.949C>T, p.Arg317Cys). *DCAF8* has been implicated in autosomal dominant Giant axonal neuropathy type 2 (OMIM # 610100). While giant axonal neuropathy type 1 is associated with a neurogenic bladder phenotype,^[^ ^27^ ^]^ the *DCAF8‐*associated type 2 phenotype is associated with distal sensory impairment, lower extremity muscle weakness, and atrophy after the second decade, but does not reference neurogenic bladder.^[^ ^28^ ^]^

One individual had a previously reported heterozygous variant in the *ENAM* gene, (ENST00000396073.3: c.1259_1260insAG, p.Pro422ValfsTer27), associated with Amelogenesis imperfecta (AI) ‐ hypoplastic autosomal dominant. AI usually occurs alone but has been reported in association with multiorgan syndromes such as nephrocalcinosis, hypothalamo–hypophyseal insufficiency, and Kohlschutter Syndrome.^[^ ^29^ ^]^ Rarely, AI has been associated with distal renal tubular acidosis, characterized by hypokalemia, systemic acidosis, and polyuria.^[^ ^30^, ^31^ ^]^

The *SEC63* variant seen in an affected individual (ENST00000369002.4:c.1605dupA, p.Pro536ThrfsTer24) is associated with polycystic liver disease (PCLD).^[^ ^32^ ^]^ However, renal features are not common in PCLD and any relationship of *SEC63* to the IC/BPS phenotype in this individual is unclear.^[^ ^33^ ^]^ Additional variants in genes with known expression in urogenital tissues and previously reported pathogenic variant interpretations are listed in **Table** **3**, with single heterozygous variants in autosomal recessive conditions reported in **Table** **4**. Individuals with rare variants in the IC/BPS candidate gene frizzled class receptor 8 *(FZD8)* are reported in **Table** **5**.

**Table 3:** Autosomal dominant Mendelian disorder‐associated variants in 109 individuals with IC/BPS

Gene	Condition	Location	Change	dbsnp	Number affected, number of families	ACMG classification	ClinVar classification (Number entries for each classification, accessed Dec 30, 2021)
*DCAF8*	Axonal hereditary motor and sensory neuropathy (HMSN2) with infrequent giant axons	chr1:160206935	ENST00000368073.3: c.949C>T, p.Arg317Cys	rs587777425	1;1	VUS/likely pathogenic PP5 Moderate, PM2, PP3 Supporting	Pathogenic (1)
*ENAM*	Amelogenesis imperfecta ‐ hypoplastic autosomal dominant ‐ local	chr4:71508403	ENST00000396073.3: c.1259_1260insAG, p.Pro422ValfsTer27)	rs587776588	1;1	Pathogenic PVS1 Very Strong, PM2 Moderate, PP5 Supporting	Pathogenic (3), Likely Pathogenic (1)
SEC63	Polycystic Liver Disease	chr6:108214755	ENST00000369002.4: c.1605dupA, p.Pro536ThrfsTer24	rs752868449	1;1	Pathogenic PVS1 Very Strong, PP3, PP5 Supporting	Pathogenic (1)

**Table 4:** Carrier status for autosomal recessive Mendelian disorders

Gene	Condition	Location	Change	dbSNP	Number affected; number of families	ACMG classification	ClinVar classification (Number entries for each classification, accessed Dec 30, 2021)
WNK1	]Neuropathy, Hereditary Sensory And Autonomic, Type IIa, Carrier	chr12:977863	NM_001184985.2: c.2971C>T, p.Arg991Ter	rs111033591	1;1	Pathogenic PVS1 Very Strong, PP5 Strong, PM2 Moderate, PP3 Supporting	Pathogenic (1)
GNE	GNE Myopathy, carrier	chr9:36218221	ENST00000372330.3: c.250G>T, p.Gly84Cys	rs62541771	1;1	Pathogenic PP5 Very Strong, PM5 Moderate, PM1, PM2, PP3 Supporting	Pathogenic (14)
ATP7B	Wilson Disease, carrier	chr13:52524268	ENST00000242839.4: c.2605G>A, p.Gly869Arg	rs191312027	6;5	Pathogenic PVS1 Very Strong, PM2 Strong, PP3 Supporting	Pathogenic (7), Likely Pathogenic (5), VUS (3)

**Table 5:** FZD8 variants in the IC/BPS cohort

Gene	Condition	Location	Change	dbSNP	Number affected; number of families	ACMG classification	ClinVar classification (Number entries for each classification, accessed July 30, 2023)	Allele frequency, gnomAD (accessed July 30, 2022)
FZD8	Candidate, interstitial cystitis/bladder pain syndrome^[^ ^51^, ^52^ ^]^	chr10:35929790	ENST00000374694.1:c.566_568delGGCinsAGT, p.Arg189_Pro190delinsLysSer		1;1	Variant of Uncertain Significance, PM2 Moderate, PP2 Supporting	N/A	0
FZD8	Candidate, interstitial cystitis/bladder pain syndrome^[^ ^51^, ^52^ ^]^	10:35929561	ENST00000374694.1:c.797A>C, p.Asn266Thr		1;1	Variant of Uncertain Significance, PM2 Moderate, PP2 Supporting	N/A	

### 3.3 Statistical Analysis

Using SKAT, the five candidate genes from the Mendelian analysis with autosomal dominant or incomplete penetrance inheritance patterns (*ATP2C1, ATP2A2*, *SIX5*, *ENAM*, and *DCAF8)* were compared between cases (*N* = 86) and controls (*N* = 90). (Table S2, Supporting Information). *ATP2C1* had a trend toward significance (*p* = 0.03, OR = 6.76) (**Table** **6**), though after Bonferroni correction only a *p*‐value of 0.01 would be considered significant. Six rare variants in *ATP2C1* were identified in the IC/BPS cohort (see Tables S2 and S3, Supporting Information). One *ATP2C1* variant was identified in the control cohort (NM_001199180.2):c.721A>G (p.Asn241Asp), scored as a VUS using ACMG criteria. No other candidate genes or housekeeping genes tested approached significance (see Tables S4 and S5, Supporting Information).

**Table 6:** SKAT analysis of Mendelian disorder‐associated genes returned to families and genes with plausible mechanisms of causing IC/BPS (86 cases and 90 controls). Markers and additional details are available in Tables S2 and S3, Supporting Information

Gene	Markers	*p*‐value (under 0.01 considered significant)	OR
*ATP2A2*	1	0.73	0.51
*DCAF8*	1	0.83	1.06
*ENAM*	6	0.19	3.34
*SIX5*	5	0.2	2.2
*ATP2C1*	6	0.03	6.76

## 4 Discussion

The etiology of IC/BPS continues to remain elusive, However, first degree relatives of individuals with IC/BPS have an increased risk of also having an IC/BPS diagnosis.^[^ ^34^ ^]^ Recent studies of kidney and urologic disorders have also shown that many individuals have genetic diseases that are undiagnosed based on standard clinical evaluation.^[^ ^8^, ^9^, ^10^, ^11^, ^12^ ^]^ In particular, 5–8% of individuals with developmental disorders of the urinary tract have an undetected genomic disorder.^[^ ^13^, ^14^, ^15^ ^]^ Therefore, we hypothesized that a Mendelian disorder or a pathway of related disorders could be causative of symptoms diagnosed as IC/BPS.

Our analysis utilized a Mendelian approach to analyze the ES data from 109 individuals with IC/BPS. This would allow us to both identify individuals with a potential mis‐diagnosis of IC/BPS and work to identify an IC/BPS candidate gene or pathway of genes. In IC/BPS probands, we identified rare variants in two bladder‐expressed genes in the ATP‐family ‐ *ATP2A2* and *ATP2C1*.^[^ ^35^ ^]^ Pathogenic variants in these genes cause two related skin disorders called Darier‐White and Hailey‐Hailey disease, respectively. Individuals with these disorders present with vesicular or blistering skin lesions that can affect the genitourinary areas and cause pain (**Table** **7**). The clinical follow‐up of the affected individuals with *ATP2A2* and *ATP2C1* gene variants demonstrated that they all have skin findings consistent with Darier‐White and Hailey‐Hailey disease. These findings suggested that molecular defects that primarily manifest in skin and mucosal epithelial injury may also participate in the pathogenesis of urothelial defects and IC/BPS.

**Table 7:** Comparative examination of the pathology and expression of genetic disorders caused by identified candidate genes (*ATP2C1*, *ATP2A2*, and *SIX5* respectively)

Symptoms	Interstitial cystitis	Hailey‐Hailey (*ATP2C1*)	Darier‐White (*ATP2A2*)	Branchiootorenal syndrome 2 (*SIX5*)
Urgency, frequency (urination)	*X*			
Pelvic/bladder pain	*X* ^[^ ^59^ ^]^			
Skin lesions		*X* ^[^ ^57^ ^]^	*X* ^[^ ^40^, ^58^ ^]^	
Vulval pain/lesions	*X* ^[^ ^59^ ^]^	*X* ^[^ ^57^ ^]^	*X* ^[^ ^58^ ^]^	
Bladder/smooth muscle involvement	*X* ^[^ ^59^ ^]^			
Abnormality of the nail		*X* ^[^ ^62^ ^]^	*X* ^[^ ^40^ ^]^	
Flare‐ups of condition	*X* ^[^ ^63^ ^]^	*X* ^[^ ^62^ ^]^	*X* ^[^ ^40^ ^]^	
Vesicoureteral reflux	*X* ^[^ ^60^ ^]^			*X* ^[^ ^61^ ^]^
Causative gene expressed in bladder		*X* ^[^ ^65^ ^]^	*X* ^[^ ^65^ ^]^	
Causative gene expressed in genital tissue				Endocervix epithelium, uterine tube epithelium^[^ ^58^, ^64^ ^]^

Darier‐White Disease, an autosomal dominant disease caused by mutations in *ATP2A2*, is a keratinizing disorder that leads to the formation of small hyperkeratotic papules in seborrheic skin areas but can also affect mucosal membranes, nails, and the genitourinary areas.^[^ ^35^, ^36^, ^37^, ^38^ ^]^ The histology shows acantholysis, hyperkeratosis, and dyskeratosis (the premature differentiation of keratinocytes).^[^ ^39^, ^40^ ^]^ We identified a likely pathogenic variant in *ATP2A2* (ENST00000539276.2: c.235G>A, p.Glu79Lys) in two probands (SF355, SF138). This variant segregates with disease in family SF355 (Figure 1). In addition to a diagnosis of IC/BPS, affected individuals in this family also report Hunner's ulcers, vulvodynia, and psoriasis. Vulvar localization and psoriasis are consistent with a diagnosis of Darier‐White disease.^[^ ^41^, ^42^ ^]^

The *ATP2A2* gene encodes the sarco(endo)plasmic reticulum calcium‐ATPase 2 (SERCA2) enzyme, which is involved in regulation of positively charged calcium atoms inside cells by pumping Ca2+ into the endoplasmic and sarcoplasmic reticulum. Altered intracellular calcium signaling may result in the abnormal epithelial development and dysregulated cell proliferation that lead to skin lesions. In addition, the SERCA pump is a major Ca2+ removal mechanism in small dorsal root ganglion (DRG) neurons.^[^ ^43^ ^]^ Lumbosacral DRG neurons mediate bladder sensation and are essential for normal voiding and pain detection. it is therefore possible that mutations in SERCA2 result in altered DRG neuron function and pain perception and also cause dysregulated cell proliferation in the urogenital tract resulting in the bladder balding and Hunner's ulcer often seen in IC/BPS. These findings suggest that further study of SERCA2 in bladder epithelium biology is warranted.

Pathogenic variants in *ATP2C1* cause Hailey‐Hailey disease, another autosomal dominant skin disorder.^[^ ^44^ ^]^ Impairment of *ATP2C1* leads to a dysfunction in the Golgi‐associated human secretory pathway Ca2+/Mn2+ ATPase (hSPCA1) that results in recurrent blisters and erosions in intertriginous sites.^[^ ^45^ ^]^ Vulvar lesions are also possible.^[^ ^44^, ^46^, ^47^ ^]^ This causes pain, vulvodynia, pruritus, and the development of chronic malodorous abnormal growths at risk of infection with *Staphylococcus aureus* and *Candida albicans*. The symptoms are typically exacerbated with sweating, friction, and heat. We identified two private heterozygous variants in *ATP2C1* in two unrelated individuals with IC/BPS. The SKAT analysis indicated that 6 additional individuals in our IC/BPS cohort have rare *ATP2C1* missense variants, conferring an OR of 6.76 (See Table 6 and Tables S2 and S3, Supporting Information). It is possible that hypomorphic variants in *ATP2C1* increase the risk of intertriginous erosion and result in the IC/BPS phenotype. Studies show that knockdown of SPCA1 results in disruption of Golgi morphology in HeLa cells,^[^ ^48^, ^49^, ^50^ ^]^ and reduction of the amount of Ca^2+^ stored in the Golgi lumen.^[^ ^49^, ^50^, ^51^ ^]^ The Golgi complex is tightly integrated into the urothelial cellular system, where it is crucial for the health of the blood–urine barrier, mainly through its association with uroplakins.^[^ ^49^ ^]^ One can hypothesize that mutations of *ATP2C1* may also result in a disruption of Golgi morphology in urothelial tissue, impairing the formation of the blood‐urine barrier, a hallmark of IC/BPS.^[^ ^52^ ^]^

We also identified a variant in the *SIX5* gene (ENST00000317578.6:c.472G>A, p.Ala158Thr) known to cause BOR2 syndrome in one of our families with 3 generations of individuals with IC/BPS. Another family member has a bladder malformation. As BOR2 syndrome is known to have incomplete penetrance and associated kidney and lower genitourinary anomalies, the IC/PBS symptomology is potentially a mild manifestation of this genitourinary malformation syndrome.

Two individuals with IC/BPS had rare variants in *FZD8* (Table 5), which is a known candidate biomarker for IC/BPS.^[^ ^53^, ^54^, ^55^, ^56^ ^]^ Keay et al. identified a glycosylated frizzled‐related peptide inhibitor of bladder cell proliferation that is secreted specifically by bladder epithelial cells from affected individuals.^[^ ^53^, ^55^ ^]^ This peptide inhibitor has homology to the putative sixth transmembrane domain of frizzled‐8.^[^ ^56^ ^]^ It is possible that rare variations in *FZD8* are contributory to the IC/BPS phenotype in these individuals, though much more investigation into the potential impact of this gene in a clinical syndrome.

Limitations of this study include that our sample size is small. Therefore, we did not have the power necessary to run a genome‐wide SKAT analysis and had to restrict the gene list to a small number of candidates. Additionally, we did not query structural variation (SV) or copy number variation (CNV). To extend these findings, we are attempting to enroll additional affected family members to screen them for the candidate genes discovered in this study. Moreover, we plan to follow the probands with *ATP2C1* and *ATP2A2* variants to see if interventions targeting their skin disorders will also ameliorate their IC/BPS symptoms. Future studies should also engage more family members and include parental ES and CNV assessment so that *de novo* versus inherited status can be determined, and ultimately future mechanistic studies will allow us to understand the role that *ATP2C1* and *ATP2A2* may play in IC/BPS. That said, it is also possible that the *ATP2C1* and *ATP2A2* variants are coincidental, and the individuals with these variants have co‐occurring but non‐overlapping disorders.

In conclusion, we have identified variants in the genes *SIX5*, *ATP2C1*, and *ATP2A2* that appear to explain aspects of the IC/BPS phenotype in affected individuals. As IC/BPS is a diagnosis of exclusion, we aim to use genetics to stratify the cohort, identifying families with overlapping phenotypic diagnoses like the *SIX5* family. We hope this will allow us to finally discover a genetic cause of IC/PBS, which will lead to faster definitive diagnostics and genetic counseling while opening the door to more targeted therapeutics for this long‐neglected disorder.

## Conflict of Interest

The authors declare no conflict of interest.

## Author Contributions

E.E., M.T., A.G.G., L.K., and C.A.B. contributed to the conceptualization and primary writing. E.E., M.T., P.S., J.P., V.Z., R.N.Y., S.B., C.B., P.B.A., A.H.B., and C.A.B. contributed to data collection, analysis and interpretation of results, discussion, review, and editing of the manuscript.

## Supporting information

Supporting Information

Supplemental Table 1

Supplemental Table 2

Supplemental Table 3

## Data Availability

The data that support the findings of this study are available on request from the corresponding author. The data are not publicly available due to privacy or ethical restrictions.

## References

[1] J. Q. Clemens , R. T. Meenan , M. C. O. Rosetti , S. Y. Gao , E. A. Calhoun , J. Urol. 2005, 173, 98. 15592041 10.1097/01.ju.0000146114.53828.82

[2] S. H. Berry , M. N. Elliott , M. Suttorp , L. M. Bogart , M. A. Stoto , P. Eggers , L. Nyberg , J. Q. Clemens , J. Urol. 2011, 186, 540. 21683389 10.1016/j.juro.2011.03.132 PMC3513327

[3] A. M. Suskind , S. H. Berry , B. A. Ewing , M. N. Elliott , M. J. Suttorp , J. Q. Clemens , J. Urol. 2013, 189, 141. 23164386 10.1016/j.juro.2012.08.088 PMC3894747

[4] K. E. Watkins , N. Eberhart , L. Hilton , M. J. Suttorp , K. A. Hepner , J. Q. Clemens , S. H. Berry , Gen. Hosp. Psychiatry 2011, 33, 143. 21596207 10.1016/j.genhosppsych.2011.01.004 PMC3099040

[5] H. B. Goldstein , P. Safaeian , K. Garrod , P. S. Finamore , S. Kellogg‐Spadt , K. E. Whitmore , Int. Urogynecol. J. 2008, 19, 1683. 10.1007/s00192-008-0712-x 18766291

[6] I. Offiah , S. B. Mcmahon , B. A. O'Reilly , Int. Urogynecol. J. 2013, 24, 1243. 23430074 10.1007/s00192-013-2057-3

[7] C. K. Payne , G. F. Joyce , M. Wise , J. Q. Clemens , J. Urol. 2007, 177, 2042. 17509284 10.1016/j.juro.2007.01.124

[8] E. E. Groopman , M. Marasa , S. Cameron‐Christie , S. Petrovski , V. S. Aggarwal , H. Milo‐Rasouly , Y. Li , J. Zhang , J. Nestor , P. Krithivasan , W. Y. Lam , A. Mitrotti , S. Piva , B. H. Kil , D. Chatterjee , R. Reingold , D. Bradbury , M. Divecchia , H. Snyder , X. Mu , K. Mehl , O. Balderes , D. A. Fasel , C. Weng , J. Radhakrishnan , P. Canetta , G. B. Appel , A. S. Bomback , W. Ahn , N. S. Uy , et al., N. Engl. J. Med. 2019, 380, 142. 30586318 10.1056/NEJMoa1806891 PMC6510541

[9] S. Lata , M. Marasa , Y. Li , D. A. Fasel , E. Groopman , V. Jobanputra , H. Rasouly , A. Mitrotti , R. Westland , M. Verbitsky , J. Nestor , L. M. Slater , V. D'agati , M. Zaniew , A. Materna‐Kiryluk , F. Lugani , G. Caridi , L. Rampoldi , A. Mattoo , C. A. Newton , M. K. Rao , J. Radhakrishnan , W. Ahn , P. A. Canetta , A. S. Bomback , G. B. Appel , C. Antignac , G. S. Markowitz , C. K. Garcia , K. Kiryluk , et al., Ann. Intern. Med. 2018, 168, 100. 29204651 10.7326/M17-1319 PMC5947852

[10] S. Sanna‐Cherchi , K. Kiryluk , K. E. Burgess , M. Bodria , M. G. Sampson , D. Hadley , S. N. Nees , M. Verbitsky , B. J. Perry , R. Sterken , V. J. Lozanovski , A. Materna‐Kiryluk , C. Barlassina , A. Kini , V. Corbani , A. Carrea , D. Somenzi , C. Murtas , N. Ristoska‐Bojkovska , C. Izzi , B. Bianco , M. Zaniew , H. Flogelova , P. L. Weng , N. Kacak , S. Giberti , M. Gigante , A. Arapovic , K. Drnasin , G. Caridi , et al., Am. J. Hum. Genet. 2012, 91, 987. 23159250 10.1016/j.ajhg.2012.10.007 PMC3516596

[11] S. Sanna‐Cherchi , R. V. Sampogna , N. Papeta , K. E. Burgess , S. N. Nees , B. J. Perry , M. Choi , M. Bodria , Y. Liu , P. L. Weng , V. J. Lozanovski , M. Verbitsky , F. Lugani , R. Sterken , N. Paragas , G. Caridi , A. Carrea , M. Dagnino , A. Materna‐Kiryluk , G. Santamaria , C. Murtas , N. Ristoska‐Bojkovska , C. Izzi , N. Kacak , B. Bianco , S. Giberti , M. Gigante , G. Piaggio , L. Gesualdo , et al., N. Engl. J. Med. 2013, 369, 621. 23862974 10.1056/NEJMoa1214479 PMC3846391

[12] S. Sanna‐Cherchi , R. Westland , G. M. Ghiggeri , A. G. Gharavi , J. Clin. Invest. 2018, 128, 4. 29293093 10.1172/JCI95300 PMC5749511

[13] J. F. Jhang , Y. H. Hsu , Y. H. Jiang , H. C. Kuo , Pain Physician 2016, 19, E581. 27228524

[14] M. Verbitsky , R. Westland , A. Perez , K. Kiryluk , Q. Liu , P. Krithivasan , A. Mitrotti , D. A. Fasel , E. Batourina , M. G. Sampson , M. Bodria , M. Werth , C. Kao , J. Martino , V. P. Capone , A. Vivante , S. Shril , B. H. Kil , M. Marasa , J. Y. Zhang , Y.‐J. Na , T. Y. Lim , D. Ahram , P. L. Weng , E. L. Heinzen , A. Carrea , G. Piaggio , L. Gesualdo , V. Manca , G. Masnata , et al., Nat. Genet. 2019, 51, 117. 30578417 10.1038/s41588-018-0281-y PMC6668343

[15] A. Vivante , M.‐J. Kleppa , J. Schulz , S. Kohl , A. Sharma , J. Chen , S. Shril , D.‐Y. Hwang , A.‐C. Weiss , M. M. Kaminski , R. Shukrun , M. J. Kemper , A. Lehnhardt , R. Beetz , S. Sanna‐Cherchi , M. Verbitsky , A. G. Gharavi , H. M. Stuart , S. A. Feather , J. A. Goodship , T. H. J. Goodship , A. S. Woolf , S. J. Westra , D. P. Doody , S. B. Bauer , R. S. Lee , R. M. Adam , W. Lu , H. M. Reutter , E. O. Kehinde , et al., Am. J. Hum. Genet. 2015, 97, 291. 26235987 10.1016/j.ajhg.2015.07.001 PMC4862256

[16] J. W. Warren , T. L. Jackson , P. Langenberg , D. J. Meyers , J. Xu , Urology 2004, 63, 17. 10.1016/j.urology.2003.08.027 14751339

[17] M. P. O'leary , G. R. Sant , F. J. Fowler , K. E. Whitmore , J. Spolarich‐Kroll , Urology 1997, 49 58. 9146003 10.1016/s0090-4295(99)80333-1

[18] F. M. E. Wagenlehner , J. W. O. Van Till , V. Magri , G. Perletti , J. G. A. Houbiers , W. Weidner , J. C. Nickel , Eur. Urol. 2013, 63, 953. 23141933 10.1016/j.eururo.2012.10.042

[19] D. A. Dyment , A. O'donnell‐Luria , P. B. Agrawal , Z. Coban Akdemir , K. A. Aleck , D. Antaki , H. Al Sharhan , P.‐Y. B. Au , H. Aydin , A. H. Beggs , K. Bilguvar , E. Boerwinkle , H. Brand , C. A. Brownstein , S. Buyske , B. Chodirker , J. Choi , A. E. Chudley , C. L. Clericuzio , G. F. Cox , C. Curry , E. Boer , B. B. A. Vries , K. Dunn , C. M. Dutmer , E. M. England , J. A. Fahrner , B. B. Geckinli , C. A. Genetti , A. Gezdirici , et al., Am. J. Med. Genet. A 2021, 185, 119. 33098347 10.1002/ajmg.a.61926 PMC8197629

[20] S. Rockowitz , N. Lecompte , M. Carmack , A. Quitadamo , L. Wang , M. Park , D. Knight , E. Sexton , L. Smith , B. Sheidley , M. Field , I. A. Holm , C. A. Brownstein , P. B. Agrawal , S. Kornetsky , A. Poduri , S. B. Snapper , A. H. Beggs , T. W. Yu , D. A. Williams , P. Sliz , npj Genomic Med. 2020, 5, 29. 10.1038/s41525-020-0137-0 PMC7338382 32655885

[21] I. A. Adzhubei , S. Schmidt , L. Peshkin , V. E. Ramensky , A. Gerasimova , P. Bork , A. S. Kondrashov , S. R. Sunyaev , Nat. Methods 2010, 7, 248. 20354512 10.1038/nmeth0410-248 PMC2855889

[22] S. Richards , N. Aziz , S. Bale , D. Bick , S. Das , J. Gastier‐Foster , W. W. Grody , M. Hegde , E. Lyon , E. Spector , K. Voelkerding , H. L. Rehm , Genet. Med. 2015, 17, 405. 25741868 10.1038/gim.2015.30 PMC4544753

[23] I. Ionita‐Laza , S. Lee , V. Makarov , J. D. Buxbaum , X. Lin , Am. J. Hum. Genet. 2013, 92, 841. 23684009 10.1016/j.ajhg.2013.04.015 PMC3675243

[24] S. Rockowitz , N. Lecompte , M. Carmack , A. Quitadamo , L. Wang , M. Park , D. Knight , E. Sexton , L. Smith , B. Sheidley , M. Field , I. A. Holm , C. A. Brownstein , P. B. Agrawal , S. Kornetsky , A. Poduri , S. B. Snapper , A. H. Beggs , T. W. Yu , D. A. Williams , P. Sliz , NPJ Genom. Med. 2020, 5, 29. 32655885 10.1038/s41525-020-0137-0 PMC7338382

[25] E. Eisenberg , E. Y. Levanon , Trends Genet. 2013, 29, 569. 23810203 10.1016/j.tig.2013.05.010

[26] A. Siepel , G. Bejerano , J. S. Pedersen , A. S. Hinrichs , M. Hou , K. Rosenbloom , H. Clawson , J. Spieth , L. W. Hillier , S. Richards , G. M. Weinstock , R. K. Wilson , R. A. Gibbs , W. J. Kent , W. Miller , D. Haussler , Genome Res. 2005, 15, 1034. 16024819 10.1101/gr.3715005 PMC1182216

[27] M. Akagi , I. Mohri , Y. Iwatani , K. Kagitani‐Shimono , T. Okinaga , N. Sakai , K. Ozono , M. Taniike , Brain Dev. 2012, 34, 156. 21356581 10.1016/j.braindev.2011.02.003

[28] C. J. Klein , Y. Wu , P. Vogel , H. H. Goebel , C. Bonnemann , K. Zukosky , M.‐V. Botuyan , X. Duan , S. Middha , E. J. Atkinson , G. Mer , P. J. Dyck , Neurology 2014, 82, 873. 24500646 10.1212/WNL.0000000000000206 PMC3959756

[29] Z. Kirzioglu , K.‐G. Ulu , M.‐T. Sezer , S. Yuksel , Med. Oral Patol. Oral Cir. Bucal 2009, 14, e579. 19680205 10.4317/medoral.14.e579

[30] P. Reddy , S. Aravelli , S. Goud , L. Malathi , Cureus 2019, 11, e5060. 31516772 10.7759/cureus.5060 PMC6721873

[31] R. Misgar , Z. Hassan , A. Wani , M. Bashir , Indian J. Nephrol. 2017, 27, 225. 28553046 10.4103/0971-4065.202826 PMC5434692

[32] S. Davila , L. Furu , A. G. Gharavi , X. Tian , T. Onoe , Q. Qian , A. Li , Y. Cai , P. S. Kamath , B. F. King , P. J. Azurmendi , P. Tahvanainen , H. Käriäinen , K. Höckerstedt , O. Devuyst , Y. Pirson , R. S. Martin , R. P. Lifton , E. Tahvanainen , V. E. Torres , S. Somlo , Nat. Genet. 2004, 36, 575. 15133510 10.1038/ng1357

[33] W. R. Cnossen , J. P. Drenth , Orphanet J. Rare Dis. 2014, 9, 69. 24886261 10.1186/1750-1172-9-69 PMC4030533

[34] J. W. Warren , T. L. Jackson , P. Langenberg , D. J. Meyers , J. Xu , Urology 2004, 63, 17. 10.1016/j.urology.2003.08.027 14751339

[35] R. G. Nellen , P. M. Steijlen , M. A. van Steensel , M. Vreeburg , Hum Mutat. 2017, 38, 343. 28035777 10.1002/humu.23164

[36] S. M. Burge , J. D. Wilkinson , J. Am. Acad. Dermatol. 1992, 27, 40. 1619075 10.1016/0190-9622(92)70154-8

[37] A. Takagi , M. Kamijo , S. Ikeda , J. Dermatol. 2016, 43, 275. 26945535 10.1111/1346-8138.13230

[38] A. Klausegger , M. Laimer , J. W. Bauer , Hautarzt 2013, 64, 22. 23337962 10.1007/s00105-012-2408-x

[39] S. M. Cooper , S. M. Burge , Am. J. Clin. Dermatol. 2003, 4, 97. 12553850 10.2165/00128071-200304020-00003

[40] A. Sakuntabhai , V. Ruiz‐Perez , S. Carter , N. Jacobsen , S. Burge , S. Monk , M. Smith , C. S. Munro , M. O'donovan , N. Craddock , R. Kucherlapati , J. L. Rees , M. Owen , G. M. Lathrop , A. P. Monaco , T. Strachan , A. Hovnanian , Nat. Genet. 1999, 21, 271. 10080178 10.1038/6784

[41] C. Cosso , F. Rongioletti , G. Zampogna , D. Camellino , M. Cutolo , M. A. Cimmino , Reumatismo 2013, 65, 86. 23877414 10.4081/reumatismo.2013.86

[42] C. M. Ridley , C. H. Buckley , Br. J. Obstet. Gynaecol. 1991, 98, 112. 10.1111/j.1471-0528.1991.tb10325.x 1998622

[43] D. Mandge , R. Manchanda , PLoS Comput. Biol. 2018, 14, e1006293. 30020934 10.1371/journal.pcbi.1006293 PMC6066259

[44] Y. Dai , L. Yu , Y. Wang , M. Gao , P. Wang , Front Genet. 2021, 14, 777630. 10.3389/fgene.2021.777630 PMC8712934 34970303

[45] A. Chiaravalloti , M. Payette , J. Drugs Dermatol. 2014, 13, 1254. 25607561

[46] J. S. Wieselthier , Arch. Dermatol. 1993, 129, 1344. 8215508

[47] V. Von Felbert , M. Hampl , C. Talhari , R. Engers , M. Megahed , Am. J. Obstet. Gynecol. 2010, 203, e5. 10.1016/j.ajog.2010.06.041 20816142

[48] M. Micaroni , G. Perinetti , C. P. Berrie , A. A. Mironov , Traffic 2010, 11, 1315. 20604898 10.1111/j.1600-0854.2010.01096.x

[49] M. E. Kreft , A. A. Mironov , S. Hudoklin , Histochem. Cell Biol. 2022, 158, 229. 35773494 10.1007/s00418-022-02121-0 PMC9399047

[50] V. Lissandron , P. Podini , P. Pizzo , T. Pozzan , Proc. Natl. Acad. Sci. USA 2010, 107, 9198. 20439740 10.1073/pnas.1004702107 PMC2889052

[51] K. Van Baelen , J. Vanoevelen , G. Callewaert , J. B. Parys , H. De Smedt , L. Raeymaekers , R. Rizzuto , L. Missiaen , F. Wuytack , Biochem. Biophys. Res. Commun. 2003, 306, 430. 12804581 10.1016/s0006-291x(03)00977-x

[52] J. Neuhaus , M. Berndt‐Paetz , A. Gonsior , Diagnostics 2021, 11, 2231. 34943467 10.3390/diagnostics11122231 PMC8700473

[53] S. K. Keay , C.‐O. Zhang , J. Shoenfelt , D. R. Erickson , K. Whitmore , J. W. Warren , R. Marvel , T. Chai , Urology 2001, 57, 9. 11378043 10.1016/s0090-4295(01)01127-x

[54] S. Keay , C.‐O. Zhang , J. L. Shoenfelt , T. C. Chai , Urology 2003, 61, 1278. 12809929 10.1016/s0090-4295(03)00005-0

[55] W. Yang , Y. Kim , T. K. Kim , S. K. Keay , K. P. Kim , H. Steen , M. R. Freeman , D. Hwang , J. Kim , BJU Int. 2012, 110, E1138. 22738385 10.1111/j.1464-410X.2012.11299.x PMC3461241

[56] S. K. Keay , Z. Szekely , T. P. Conrads , T. D. Veenstra , J. J. Barchi , C.‐O. Zhang , K. R. Koch , C. J. Michejda , Proc. Natl. Acad. Sci. USA 2004, 101, 11803. 15282374 10.1073/pnas.0404509101 PMC511055

[57] I. García‐Morales , L. Requena‐Caballero , R. Happle , A. Torrelo , Pediatr. Dermatol. 2018, 35, e398‐e9. 30168169 10.1111/pde.13625

[58] T. G. Salopek , A. Krol , K. Jimbow , Pediatr. Dermatol. 1993, 10, 146. 8346108 10.1111/j.1525-1470.1993.tb00042.x

[59] I. Marcu , E. C. Campian , F. F. Tu , Semin. Reprod. Med. 2018, 36, 123. 30566978 10.1055/s-0038-1676089

[60] J. E. Lee , B. H. Yi , H. K. Lee , M. H. Lee , Y. H. Kim , AJR Am. J. Roentgenol. 2015, 204, W457‐60. 25794095 10.2214/AJR.14.13108

[61] N. Morisada , K. Nozu , K. Iijima , Pediatr. Int. 2014, 56, 309. 24730701 10.1111/ped.12357

[62] R. Szigeti , R. Kellermayer , J. Invest. Dermatol. 2006, 126, 2370. 16794587 10.1038/sj.jid.5700447

[63] S. Sutcliffe , C. S. Bradley , J. Q. Clemens , A. S. James , K. S. Konkle , K. J. Kreder , H. H. Lai , S. C. Mackey , C. P. Ashe‐McNalley , L. V. Rodriguez , E. Barrell , X. Hou , N. A. Robinson , C. Mullins , S. H. Berry , Int. Urogynecol. J. 2015, 26, 1047. 25792349 10.1007/s00192-015-2652-6 PMC4489981

[64] C. Winchester , S. Robertson , T. MacLeod , K. Johnson , M. Thomas , J. Clin. Pathol. 2000, 53, 212. 10823141 10.1136/jcp.53.3.212 PMC1731149

[65] M. Safran , N. Rosen , M. Twik , R. BarShir , T. I. Stein , D. Dahary , S. Fishilevich , D. Lancet , In Practical Guide to Life Science Databases. Springer, Singapore 2021, pp. 27–56.

