# Supplementary material for: Mendelian Disorders in an Interstitial Cystitis/Bladder Pain Syndrome Cohort
Source: Adv Genet (Hoboken). 2022 Nov 27;4(1):2200013. doi: 10.1002/ggn2.202200013 (PMC10000272; doi:10.1002/ggn2.202200013)
Supplement: Supplementary file 1 — Supporting Information [file GGN2-4-2200013-s001.pdf]

ADVANCED GENETICS
Open Access

Supporting Information

for *Advanced Genetics*, DOI 10.1002/ggn2.202200013

Mendelian Disorders in an Interstitial Cystitis/Bladder Pain Syndrome Cohort

*Elicia Estrella, Shira Rockowitz, Marielle Thorne, Pressley Smith, Jeanette Petit, Veronica Zehnder, Richard N. Yu, Stuart Bauer, Charles Berde, Pankaj B. Agrawal, Alan H. Beggs, Ali G. Gharavi, Louis Kunkel and Catherine A. Brownstein**

Supplemental Table 1. Housekeeping genes

**Gene symbol**	**RefSeq accession number**	**Gene symbol**	**RefSeq accession number**
C1orf43	NM_015449	***GAPDH***	NM_002046
CHMP2A	NM_014453	***ACTB***	NM_001101
EMC7	NM_020154	***ZNF91***	NM_003430
GPI	NM_000175	***ABCF2***	NM_005692
PSMB2	NM_002794	***KIFC3***	NM_005550
DRAP1	NM_006442	***HMBS***	NM_000190
PTDSS1	NM_014754	***SDHA***	NM_004168
REEP5	NM_005669	***B2M***	NM_004048
EXTL3	NM_001440	***YWHAZ***	NM_003406
PFDN1	NM_002622	***SGTA***	NM_003021
VPS29	NM_016226	***AARS***	NM_001605
EMC7	NM_152595	***DRAP1***	NM_006442
PCGF2	NM_007144		

Supplemental Table 2. SKAT analysis results

Supplemental Table 3. ACMG Interpretation of SKAT results for IC/BPS carriers

Supplemental Table 4. SKAT analysis of housekeeping genes (86 cases and 90 controls).

Set 1

Gene	Markers	p-value (under 0.01 considered significant)	OR
SGTA	3	0.93	1.06
ACTB	1	NA	0.33
ABCF2	2	0.37	3.2
YWHAZ	NA		
B2M	NA		

Set 2

Gene	Markers	p-value (under 0.01 considered significant)	OR
EMC7	5	0.07	0.21
SUMO3	2	0.63	1.04
SDHA	6	0.06	0.25
PFDN1	1	NA	
CHMP2A	NA		

Set 3

Gene	Markers	p-value (under 0.01 considered significant)	OR
GAPDH	4	0.03	0.34
KIFC3	10	0.5	0.57
AARS	10	0.64	1.24
SGTA	3	0.93	1.03
EXTL3	2	0.15	5.4

Set 4

Gene	Markers	p-value (under 0.01 considered significant)	OR
DRAP1	2	0.37	3.18
PCGF2	2	0.6	0.35
ZNF91	7	0.36	1.6
GPI	5	0.73	1.03
VPS29	NA		

Set 5

Gene	Markers	p-value (under 0.01 considered significant)	OR
PSMB2	3	0.84	1.57
C1orf43	1	NA	1.05
HMBS	5	0.26	0.5
REEP5	3	0.05	0.11
PTDSS1	2	0.21	0.34

Supplemental Table 5. Housekeeping gene SKAT analysis results
